# Exploring the cultural effects of gender on perceptions of cutaneous leishmaniasis: a systematic literature review

**DOI:** 10.1186/s41256-022-00266-y

**Published:** 2022-09-26

**Authors:** Brianne Wenning, Helen Price, Hasara Nuwangi, Kelemework Tafere Reda, Ben Walters, Reem Ehsanullah, Greice Viana, Alina Andras, Lisa Dikomitis

**Affiliations:** 1 Kent and Medway Medical School, University of Kent and Canterbury Christ Church University, Canterbury, CT2 7FS UK; 2 School of Life Sciences, Keele University, Newcastle-under-Lyme, UK; 3 Department of Community Medicine, Faculty of Medicine and Allied Sciences, Rajarata University of Sri Lanka, Saliyapura, Sri Lanka; 4 College of Social Sciences and Languages, Mekelle University, Mekelle, Ethiopia; 5 Royal Stoke University Hospital, Stoke-on-Trent, UK; 6 School of Medicine, RCSI Bahrain, Busaiteen, Bahrain; 7 State University of Rio de Janeiro (UERJ) and Institute of Collective Health, Federal University of Bahia, Salvador, Brazil; 8 School of Medicine, Keele University, Newcastle-under-Lyme, UK

**Keywords:** Cultural dimension of health, Gender, Qualitative research, Stigma, Health-seeking behaviour, Neglected disease, Health beliefs

## Abstract

**Background:**

More than one million people each year become infected by parasites that cause the disease cutaneous leishmaniasis (CL). This disease manifests as one or more skin lesions or ulcers that are slow to heal with variable response rates to drug treatments. Thus far, little attention has been paid to how the cultural effects of gender shape perceptions and experiences of CL. This review aims to bring together and analyse existing studies which use qualitative data to explore these differences. These studies offered insights into our specific research questions.

**Methods:**

We conducted a systematic review of the literature pertaining to either CL or muco-cutaneous leishmaniasis (MCL) through EBSCO, EMBASE, Medline, Scopus and Web of Science databases. To meet inclusion criteria, articles had to be either qualitative or mixed-method with a qualitative component. They also had to include a reflection on how the gender of participants impacted the findings and addressed the lived experiences of CL. We did not exclude articles based on the language they were published in or in which country the study took place.

**Results:**

From a total of 1589 potential articles, we found that thirteen met the inclusion criteria. These articles were published in English, Spanish or Portuguese and reported on studies carried out in various countries in Africa, Asia and South America. After using the principles of a meta-ethnography to analyse these studies, we generated several key themes. We found that health-seeking behaviours, treatment choices, stigma and the impact of scarring are shaped by gender in a variety of contexts.

**Conclusions:**

Gender impacts on an individual’s experience of CL. In particular, women are more constricted in their health-seeking behaviours and experience more stigma both from the active lesions and from scarring than men. In many contexts, however, men are more at risk of becoming infected by the parasite that causes CL and may turn to more harmful or aggressive self-treatments. We recommend that future research on CL should consider the impact of gender as this can create very different experiences for individuals.

**Supplementary Information:**

The online version contains supplementary material available at 10.1186/s41256-022-00266-y.

## Background

Leishmaniasis is a group of neglected tropical diseases (NTDs) that are endemic to 98 countries around the world [1]. While we are aware the label ‘tropical’ is becoming increasingly problematic [2], we will use this term in our paper to denote that leishmaniasis belongs to a specific subset of neglected diseases. Leishmaniasis is caused by infection with a protozoan parasite, *Leishmania *spp., which is spread to humans by the bite of an infected sand fly. Though three main forms of the disease exist, approximately 75% of all cases are classified as cutaneous leishmaniasis (CL), with the other 25% comprising visceral leishmaniasis and muco-cutaneous leishmaniasis (MCL) [3]. CL is characterised by one or more skin lesions which ulcerate. These are slow to heal, with variable response rates to drug treatments, and may result in permanent and disfiguring scarring. Due to this skin pathology, CL is recognised to be a severely stigmatising skin disease [4]. Currently, there are as many as one million new cases of CL each year and the global disease burden has been estimated by the World Health Organisation (WHO) to be about 2.4 million disability-adjusted life years (DALYs), representing the highest single disease burden on the WHO list of neglected diseases [3–6]. The geographic spread of the cutaneous form is changing as climate change intensifies, allowing the sand fly vector species to spread north [7, 8].

Research suggests that people with visible skin conditions may experience increased stigma, isolation, financial hardships and impacts on their mental health [9]. Emerging evidence points to the psychosocial effects that CL in particular has on individuals. CL can have lasting effects on mental health, social status and quality of life (QoL), particularly when lesions are active [10, 11]. Recent research, however, argues that the effects of CL do not simply stop when the lesion heals, but may continue due to scarring. This scarring may have such profound mental health effects as to contribute to major depressive disorder [12]. The inclusion of inactive CL is highly significant as it has been estimated that there may be more than 40 million people living with the psychosocial effects of scarring due to CL [12].

This review explores the cultural effects of gender on perceptions of CL. It is important to distinguish gender from sex in this review. Sex refers to biological and physiological characteristics. Gender, on the other hand, is socially and culturally constructed. It describes an array of behaviours, norms, expectations and relational constructions for an individual largely based on whether they are born male or female [13–15]. By considering the culturally mediated effects of gender on the perceptions of and experiences with CL, we seek a more holistic understanding of how CL impacts on a person’s life and in which life domains.

Research into the impact of gender on psychosocial domains suggests that for most NTDs, women are disproportionately affected [11, 12, 16]. This is especially pronounced in the case of another skin condition, leprosy. In this case, women are seen as facing ‘triple jeopardy’ in terms of discrimination due to their gender, the disabilities that can result from leprosy and the impact of stigma from having the disease, all which have serious implications for their education, employment, marriage and overall participation in the society [17, 18]. It may be similar in the case of CL, where lesions are sometimes mistaken for leprosy [19–21]. A recent systematic review found that women affected by CL had a 2.13 higher chance than those without of suffering from a mental health condition such as anxiety or depression [11]. While they only included quantitative studies, it is clear that this topic warrants further investigation, particularly into the subjective experience of individuals with CL.

CL is a prevalent disease with the potential for stigmatisation and long-lasting psychological distress. Therefore, the aim of this review is to ascertain the ways in which gender impacts on the perceptions and experiences of living with active CL, inactive CL or MCL by analysing qualitative research. The sub questions are as follows:

1. In what ways does gender affect health-seeking behaviour?
2. Does the psychosocial impact of living with CL differ by gender in terms of perceptions and experiences?
3. Is stigma experienced by people with CL, and if so, in what ways does this differ between women and men?

## Methods

To guide our synthesis and establish our parameters, we employed a modified PICO (participants, item of interest, comparison group, outcome of interest) tool [22]. In this review, the comparison group is not applicable. This review has been prepared according to the Preferred Reporting Items for Systematic Reviews and Meta-Analyses (PRISMA) guidelines [23].

### Search strategy and study selection

Search terms fell into two main categories. The first category used terms related to CL. These terms included both formal nomenclature (e.g. cutaneous leishmaniasis, tegumentary leishmaniasis) and more local terms that have been reported in the literature on CL (e.g. oriental sore, chiclero’s ulcer, one year sore). The second category included terms related to qualitative studies (e.g. qualitative studies, phenomenology, lived experience). Gender did not feature as a category and was screened for in subsequent steps. We conducted a search of the following five electronic databases: EBSCO, EMBASE, Medline, Scopus and Web of Science. The full search strategy can be found in the Additional files (1, 2, 3, 4, 5, 6 and 7).

### Eligibility criteria

Studies were not limited by date of publication or by language. At each stage of the selection process, potential studies were compared against our inclusion and exclusion criteria. Inclusion criteria consisted of qualitative research studies of any kind (including mixed methods studies with a qualitative component) that focused on CL or MCL conducted anywhere in the world. Studies had to present findings in such a way that distinct experiences around gender could be obtained. Excluded studies were those that reported on visceral leishmaniasis or post kala azar dermal leishmaniasis (PKDL), studies with no qualitative research components and studies in which data pertaining to gender could not be separated.

### Critical appraisal and data extraction

Critical appraisal consisted of the Critical Appraisal Skills Programme (CASP) checklist for qualitative research [24]. This checklist appraises the quality of qualitative studies through a series of ten questions. All studies included in the review passed the critical appraisal as determined by the CASP tool.

### Synthesis

This systematic literature review employed a qualitative mode of analysis. Meta-analyses for qualitative studies rely on interpretation in which researchers compare and analyse reports [25]. For the purposes of analysing our data, we used the principles of meta-ethnography as described by Noblit and Hare [26].

Noblit and Hare [26] describe three possible ways to synthesise qualitative studies. For our studies on gender and CL, the line of argument method was used. In line of argument, individual studies offer insights into different aspects of a phenomenon; it is only by placing these studies together sequentially that the question can be answered. By using this method of analysis, we can better understand the impact of gender on a wide range of experiences of CL, ranging from knowledge of CL and health-seeking behaviours to body-image and the effects of stigma.

## Results

### Article inclusion process and characteristics

**Fig. 1:**
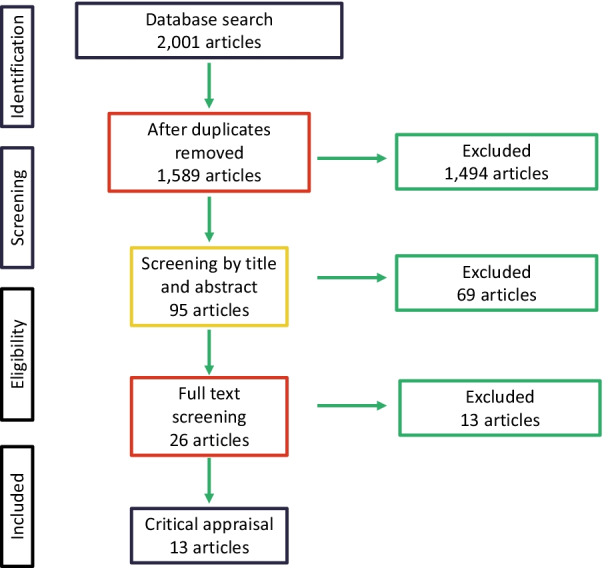
Flow diagram of the literature screening process

A total of 1589 potential studies were identified after citations and abstracts were downloaded into Mendeley [27] and duplicates removed. These studies were then uploaded into *Rayyan* software [28] where two independent reviewers (BW and RE) screened the titles and abstracts. Discrepancies were discussed with a third reviewer (BrW) and a consensus on the inclusion or exclusion of each study was reached. After this stage, a total of 95 studies remained. A full text screening of these articles by both BW and RE yielded 26 articles. During the critical appraisal and data extraction process, a further thirteen articles were identified as not meeting the inclusion criteria. This left a total of 13 articles (10 in English, 2 in Spanish and one in Portuguese) for this study. This process is described in Fig. 1.

Data extraction and critical appraisal of English-language articles were carried out by BrW, KTR and HN. Data extraction and critical appraisal of the Spanish and Portuguese-language articles was carried out by GV, HN and BrW. See Table 1 for details on the included studies.

**Table 1:** Overview of the articles included in this review

Authors	Year	Title	Country	Demographics of participants	Method	Study aim
Weigel and Armijos [19]	2001	The traditional and conventional medical treatment of cutaneous leishmaniasis in rural Ecuador	Ecuador	529 adults (336 female, 193 male) from 43 hamlets	In-depth interviews supplemented with questionnaires	To explore the knowledge, beliefs and practices regarding cutaneous leishmaniasis treatment held by an endemic population in Ecuador
Carillo-Bonilla et al. [29]	2014	Study of knowledge, attitude, and practices related to leishmaniasis: evidence of government neglect in the Colombian Darién	Colombia	252 people, 130 men and 122 women, all over the age of 15	Qualitative study through ethnography and data collection, from the perspective of Knowledge, practices and attitudes (KAP)	To understand the aspects of cutaneous leishmaniasis in populations in Colombia through studies of knowledge, practices and attitude
Bennis et al. [30]	2017	Psychosocial impact of scars due to cutaneous leishmaniasis on high school students in Errachidia province, Morocco	Morocco	Boarding school children, ages 18.1 ± 2.3 for boys and 17.2 ± 1.6 for girls	Self-administered survey with open-ended questions on the psychosocial effect of scars	To describe the psychosocial impact of CL on adolescents in Morocco’s major endemic areas
Bennis et al. [31]	2017	“The mosquitoes that destroy your face”: Social impact of cutaneous leishmaniasis in South-eastern Morocco, A qualitative study	Morocco	11 men, 29 women	Focus group	To document the psychosocial burden of CL in rural communities in Southeastern Morocco
Stewart and Brieger [32]	2009	Community views on cutaneous leishmaniasis in Istalif, Afghanistan: Implications for treatment and prevention	Afghanistan	Unknown number of respondents. Low-medium economic and education levels	Focus groups of between 6 and 9 individuals	To investigate illness recognition and treatment seeking behaviour, beliefs about etiology and transmission of the disease, and views of prevention and protection among the population of Istalif
Ramdas [33]	2012	Cruel disease, cruel medicine: Self-treatment of cutaneous leishmaniasis with harmful chemical substances in Suriname	Suriname	205 people at a Dermatology service (183 male, 22 female); 285 people from hinterland villages	Interviews with structured questionnaires, lasting between 30 min and 1 hour	To encourage health policy makers and health professionals to carefully initiate, provide and evaluate CL treatment and prevention programs
Eid et al. [34]	2019	Leishmaniasis patients’ pilgrimage to access healthcare in rural Bolivia: A qualitative study using human rights to health approach	Bolivia	14 participants, 11 male and 3 female, aged 17–50. One case of a 1 year old child where the mother was interviewed	In-depth interviews lasting between 30 and 45 minutes	To explore the experiences of patients with leishmaniasis and the challenges faced when searching for diagnosis and treatment in Bolivia using a human rights approach
Erber et al. [35]	2020	Patients’ preferences of cutaneous leishmaniasis treatment outcomes: Findings from an international qualitative study	Brazil, Burkina Faso, Colombia, Iran, Morocco, Peru, Tunisia	Males and females, median age 35	Semi-structured in-depth interviews	To assess patient-preferred outcomes for CL
Dobles-Ulloa and Perriard [36]	1994	Representation, attitudes and practices related to cutaneous leishmaniasis in people from Acosta, Costa Rica	Costa Rica	Individuals from both case and control households	48 open interviews, lasting an average of 45 min	To understand the representations, attitudes and practices about tegumentary leishmaniasis in the province of Acosta Catón de San José (Costa Rica), from the point of view of the population affected or at risk of being affected
Ramdas [37]	2016	Nuancing stigma through ethnography: The case of cutaneous leishmaniasis in Suriname	Suriname	205 people at a Dermatology service (183 male, 22 female, 81% age 20–49, 77% of whom lived in the capital city or surrounding districts) and 321 from hinterland villages (188 men, 133 females from Maroon and Indigenous communities)	Short structured interviews, 30 min - 1 hour duration	To establish perceptions and explanations for health seeking behaviour and to determine whether stigma around CL exists within Suriname
da Silva and Lopes [38]	2004	American tegumentary leishmaniasis in the perspective of who lives it	Brazil	Eight women who presented sequelae due to CL lesions, and who were being treated at a public health institution	Interviews with a phenomenological approach	To understand the perception of the body of the women affected by cutaneous leishmaniasis
Hamdam [39]	2020	Why does leishmaniasis result in life-long scars for women in Afghanistan?	Afghanistan	Male and female healthcare providers within the clinics	Focus groups and in-depth interviews	To explore the reasons behind the late presentation of female patients with leishmaniasis to the (leishmaniasis) clinic within the NMLCP of the Ministry of Public Health
Reithinger et al. [40]	2005	Social impact of leishmaniasis, Afghanistan	Afghanistan	For the survey: the most senior, available family member and focus groups	252 individuals during the house-to-house survey, 108 women across the focus groups	To establish the knowledge of CL held by residents of Kabul, Afghanistan

The data extracted from the papers included eleven main domains. These are as follows: study setting, timeframe of research, demographics of participants, data collection period, method of data collection, stigma and type, psychosocial domains, economic impact, health seeking behaviour, impacts of inactive CL (scars) and limitations of the study. All reviewers agreed that these domains were sufficient to capture the data that we were interested in from each study.

Based on the 13 articles included in our review, we identified six main themes that offered a perspective on gender: knowledge around CL, healthcare seeking, treatment types, stigma, scars and psychosocial domains. These main areas can be mapped onto our original three sub questions.

### Nomenclature of CL

Before exploring the factors around identifying and seeking treatment for the disease, it is important to establish people’s knowledge base of CL. General knowledge about CL, including insect vectors, mode(s) of transmission, disease recognition, risk factors and prevention methods, varied greatly across countries. Even the name used to label CL was wide-ranging. While a few studies reported that participants were familiar with *Leishmania* or leishmaniasis, notably those in Colombia and Morocco [29–31], many used local names which can be categorised as follows: (a) by appearance, such as *sarna brava* or ‘angry sore’ (Ecuador)[19]; (2) by duration as *saldana* or ‘one year sore’ (Afghanistan) [32]; (3) by perceived vector as is the case in ‘mosquito sore’ or ‘mosquito scar’ (Morocco) [31]; (4) by the location where the disease is endemic. This can be a specific geographical place, such as ‘sore of Touroug’ or ‘boil of Ata’, both of which are towns in Morocco [30, 31] or a more general place as is the case of *bussi-yassi* in Suriname, where *buss*i refers to the bush or forest [33], or the ‘seal of the jungle’ in Colombia [29]. Other local names include *bejuco, yatevi* [29], *espudia* and *jadyeye* [34] (from Colombia and Bolivia, respectively). Authors of these studies, however, do not indicate from where these names originate. It is only the study by Carillo-Bonino et al. [29] in which any difference in gender terminology emerged. They note that where they conducted research in Colombia, men use *bejuco* and women *yatevi* most often.

### Identifying vectors and CL

Though few individuals possessed full, accurate knowledge of key areas of CL, only one difference with regards to gender was reported in the studies when it came to aspects such as the mode of transmission and prevention. This difference occurred in the named vector for CL. The disease is transmitted by the bite of infected sand flies (genera *Phlebotomus* and *Lutzomyia*) which are less than 3 mm in length. Amongst participants in focus groups held in Afghanistan, a variety of local insects were identified as vectors for CL [32]. However, in the focus groups, the men described the sandfly responsible for CL as one which has two wings and two legs yet is so small that it is difficult to see. The women, by contrast, described the CL vector as a large insect with long wings. It was said to be bigger in size than even the malaria mosquito and had a green colour [32].

Additional differences around gender occurred in what study participants perceived to be true regarding CL. While disease recognition remained largely the same between men and women throughout the studies, one difference occurred amongst the men and women taking part in focus groups in Afghanistan. In the study by Stewart and Brieger [32], women discussed additional symptoms of CL that the men did not. Both groups described the developing lesion as small and red, resembling a pimple, that itched and grew in size. Women, however, distinguished this manifestation from another form of CL which began as a localised rash on the body, characterised by multiple lesions with a hard core, before spreading more widely [32]. Words such as ‘seeping’ and ‘contagious’ distinguished this presentation of CL.

A further gender difference in disease recognition stemmed not from the identifiable symptoms, but from the perceived gender of the lesion itself. In Colombia, the lesions were identified as either a ‘male whistle’ or a ‘female whistle’ [29]. The authors noted that the characterisation of these lesions applied to the gendered roles for men and women in that particular culture. For instance, the ‘female whistle’ was painless, yet it grew quickly and ‘cries’ – by which they meant the lesion appeared wet with discharge from the wound – and healed relatively easily. By contrast, the ‘male whistle’ did not grow visibly across the skin but instead penetrated deeper. Though much smaller in appearance, it was difficult to cure [29].

### Risk and CL

While these small, rather isolated differences were mentioned in a few of the included studies, local understandings surrounding the risk of acquiring CL and gender appeared to be more widespread. These revolve around understandings of risk in transmission, who is most at risk, what is a risky environment and who is responsible for mitigating these risks. The perceived risk of acquiring CL primarily seemed to stem from occupation and recreational activities, which often have a gender connotation. Two of the studies specifically mentioned men as at risk of contracting CL, and two studies specifically mentioned women as at risk of contracting the disease.

Research conducted in Suriname [33] and Colombia [35] indicated that men were more likely to report a CL infection. In Suriname, risk taking was associated with masculinity [33]. This extended to working in what was considered the risky and masculine sectors of gold mining, lumber and construction work. Because these occupations often took place deep in the hinterland – which participants already linked with CL – those employed in these sectors, men, were perceived as most risk and indeed were more likely to report experiences with CL.

The study in Colombia echoed this link between occupation and risk. There, CL was associated with serving in the army. This study included a total of twenty study participants from Colombia, of which 18 were men [35]. Those in this study recognised that they were at risk and, in fact, mentioned having seen many of their army colleagues becoming reinfected with CL multiple times. The link between occupation and CL risk was not necessarily overtly associated with masculinity, but as it was predominately men in this group of participants, we analysed this as predominantly pertaining to men.

While certain occupations appeared to place men at more risk amongst participants in Suriname and Colombia, delegation of domestic chores was the driving force behind placing women at greater risk in Morocco. Participants reported that women were more exposed to risk because of the farming activities in which they engaged [31]. For instance, women take care of the cattle, which includes handling manure and waste. This exposes women to more bites from the sand fly and thus increases risk of CL infection. Participants also indicated that children and tourists may be at greater risk of CL as well, but this was attributed more to their lack of acquired immunity rather than their daily activities [31].

Another study indicated risk of CL infection based on gender. In Afghanistan, women – along with children – were perceived as being more at risk [32]. The authors, however, do not elaborate on this perception. They do note that women were more likely than men to associate an unclean environment with CL. The authors explain this is because a series of educational seminars had taken place in the study area linking sanitation and CL [32]. Women indicated that housing issues were the biggest risk factor for contracting CL. These issues included lack of windows, lack of doors and the burning of homes by the Taliban [32].

While several studies reported on links between gender and risk of CL, one offered a nuanced understanding of risk. For participants in this study in Colombia, risk was associated with responsibility [29]. Acknowledging a risky environment, then, carried the responsibility of ensuring that oneself – and those in the immediate environment such as children– were sufficiently protected. For instance, for every 15 women who recognised the jungle as a risky environment, only one man did so [29]. This meant that, as reported in this study, few men took preventive measures against CL whilst in the jungle. On the other hand, three men for every one woman claimed that inside the home itself constituted a risky environment regarding CL. The authors conceptualised ‘home’ as a place of risk and strongly associated it with gender roles. For participants in this study, it meant that they neglected to fulfil the societal role of keeping a clean and safe home for their families. This sentiment was echoed in a study on CL in Costa Rica where women were responsible for controlling CL in the domestic sphere [36]. The inability to protect one’s family from CL, particularly children, was associated with a lack of care and subsequent disorder, leading many women to deny risk in the domestic space.

### Knowledge around CL

In the studies included in our systematic literature review, the cultural effects on gender and perception could be seen when it came to knowledge around CL (such as naming, vector, transmission, etc.) and appeared to be highly specific to the local context. Because of this, the literature does not currently support the notion that knowledge of CL varies according to gender in a general sense. In terms of risk, however, several studies reported that occupational pursuits or domestic responsibilities, often demarcated according to gender roles in that cultural context, increased the risk of acquiring CL. These findings, however, were not uniform as some studies indicated men were more likely to develop CL while others indicated women and, by extension, children.

### Recognising CL

Recognition of a lesion due to CL did not differ by gender. In fact, some study participants compared the early signs of CL to acne [31] or as being similar to cuts or marks from farming activities or daily life [34], meaning they did not feel compelled to seek out treatment immediately. Information on any observed behavioural differences in how men or women treat CL lesions was absent in the included studies. While it was women who cared for the wound on others, the initial recognition and the decision to seek treatment for CL did not appear to be influenced by gender [36].

#### Barriers to treatment

Diagnosis of CL typically requires microscopy to detect the parasites either through skin scraping or lesion biopsy and are often done in specialist clinics. After a positive diagnosis, biomedical treatment consists of multiple daily painful injections with a drug (pentavalent antimonial) which is toxic and can cause side effects. This treatment requires the individual to stay in a healthcare setting for this treatment for approximately 20 days, meaning they are unable to fulfil work- and domestic-related duties during this time. Given these factors, it was found that even after individuals decided to seek treatment for CL lesions, significant barriers remained. Many of these crossed gender divides and included the high economic costs of seeking treatment, the often difficult or extensive travel involved in reaching a healthcare facility and the general mistrust of Western medicine and/or the health system in general [29, 33, 34, 37]. However, across several articles included in this review, one barrier emerged specifically for women. This barrier was socially appropriate access to healthcare.

Multiple articles highlighted the gendered disparity in accessing healthcare. For instance, in Colombia, women avoided seeking treatment in local formal health settings due to a high distrust in the health system, particularly as it related to the diagnosis and treatment of CL [29]. For this reason, those with greater financial resources preferred to travel to a hospital in the urban area to receive an accurate diagnosis. Those with less resources at their disposal turned firstly to traditional medicine. Women in Brazil similarly claimed to avoid hospitals and clinics where possible. They felt apprehensive about the treatment and feared venepuncture in particular. Furthermore, they perceived the healthcare professionals administering the treatment as ‘distant’, which enhanced their overall negative perceptions of this treatment encounter [38]. Because of this, many women dropped out of treatment early. In Ecuador, accessing healthcare was only a barrier for a certain group of women: those who were pregnant or nursing, and who were consequently dissuaded from seeking treatment for the lesion due to the toxicity of the drug used and the potential for damage to the baby [19].

A further two studies conducted in Afghanistan, however, focused more in-depth on barriers to accessing formal healthcare based on gender [32, 39]. The cultural customs prevented many women from seeking healthcare treatments because they needed to be accompanied by a man, usually their husband, who often could simply not afford to leave his job to take his wife to the clinic [32]. Secondly, in accordance with culturally appropriate norms, women should only be treated by other female healthcare professionals. If female doctors and healthcare staff were not present, then women simply avoided seeking care [32, 39]. This was a significant barrier as many healthcare settings in Afghanistan faced a shortage of female healthcare workers, which created significant problems [39].

### Treating CL

Treatment options for CL varied and depended on a variety of socioeconomic, cultural and personal factors. While participants of both genders preferred biomedical treatment in one of the studies in Afghanistan [40], the authors of a second study asserted that women were more likely than men to mention natural and herbal treatments for CL [32]. Other studies are more explicit. For instance, in the Colombian study, it was noteworthy that for every man who received a biomedical treatment (meglumine antimony injections in this case), there were thirteen women who sought home remedies with plants, ointments or burning the lesions with a spoon or other hot metal [29].

Homemade and herbal treatments were explicitly mentioned in over half (10) of the studies. Often these consisted of leaves, sap or flowers of a variety of plants and herbs [19, 33], herbs such as eucalyptus, aloe vera, tea [30], or food stuffs like olive oil, eggs, honey, onion, garlic [30], salt, vegetables and green lentils [32]. These treatments, however, are described by both men and women, making it difficult to discern if or how culture impacts on treatment preference along gender lines. The only exception to this is the study from Colombia described above [29]. More aggressive forms of self-treatment, however, were not mentioned equally.

Self-treatment using harsh chemicals or extreme heat was much more likely to be discussed by men in the studies. Chemicals included lead, gasoline, bleach, household insecticides, battery acid and herbicides. The most common chemical used in this study, however, was Smeerex, a larvicide frequently used on animals. In Ecuador, men were twice as likely to believe that such treatments indicated they were more effective in healing the lesion [19]. In Afghanistan, men stated that the chemicals inside batteries could be used for treatment [32]. In Suriname, 45 of the 48 participants who used harmful, non-biomedical chemical products to treat CL were men [33]. One participant remarked on the perceived efficacy of this treatment, stating the belief that because these products are powerful and burn through everything, can also kill the sore [33]. All of these chemicals were readily found in the immediate environment, either through one’s occupational or domestic space and thus readily accessible to use.

The authors of these studies offer another reason for these harsher self-treatments amongst men. These treatments were intimately tied to notions of masculinity. In Ecuador, it was assumed that men and boys were able to tolerate these methods because they were seen as being stronger physically and emotionally [19]. Similarly, in Suriname, notions of courage and its supposed link with masculinity meant that a man must have courage if he used one of these harsh, painful methods.

### Stigma, scars and the psychosocial domains of CL

Stigma and its effects due to CL appear in the findings of several of the included studies. However, CL-associated stigma seems to vary across different cultures and contexts. It also appears to affect men and women differently, with women bearing the major burden. Furthermore, in the studies, stigma was discussed in relation to a person’s CL lesion or scar. We have chosen to discuss stigma, scars and psychosocial domains of CL together as they were often interwoven in participant’s narratives and in the author’s discussions. Table 2 contains examples of how they were presented narratively within the articles.

**Table 2:** Direct quotes form participants in the studies on the effect of CL

Theme	Quote	Country	Article
Marriage	‘She [woman with a CL scar] will be afraid about her future especially for the wedding. Meanwhile, in our society, an affected boy remains a man. There is no harm if he has scars.’	Morocco	31
	‘Well I got married at 17 and I got divorced because of this issue. He told me something about my face and I finally got separated when we were engaged. [...] I got married again, and my husband’s skin is white without any spots. I had to use creams since I was a kid. [...] Yeah, I married twice and I didn’t have a wedding ceremony both times due to my CL. [...] Because they took films at weddings and the camera takes a close shot from your face and I didn’t want that... ‘	Iran	35
Beauty	‘For a girl when the disease leaves a mark on the face for example, the girl will think that is dangerous for her beauty, which will influence her psychological state. Especially in our traditional society that is absolutely not lenient towards those who have spots on the face because they think it is hereditary.’	Morocco	31
	Women ‘are disgraced with this disease.’	Afghanistan	32
	‘It will cause their faces to be disfigured.’		
Scars	‘The scar is a mark of shame and contempt.’	Morocco	30
	‘The psychological state of the affected person can worsen after receiving treatment because the problem is that scars never disappear [even after treatment].’	Morocco	30
Psychosocial effects	‘She [a girl with CL] will have depression, a durable fear, and shame. She does not have the absolute courage to sit with her friends for fear of their mockery.’	Morocco	30
	‘A catastrophe [...] in my life named leishmaniasis, I swear. I can’t talk. I’m thinking how my leg could be amputated. How my life will look like after amputating my leg, I think about suicide, at the same time I think about my daughter how she will calm down after my death. I can’t live without my leg.’	Tunisia	35

Notions of beauty and marriage impacted on those affected by CL. Women were, however, disproportionately affected by stigma in these domains [30–32, 40]. While both men and women were affected by disfigurement, men frequently talked about what they perceived as the impact on women [32]. In Afghanistan, for instance, men assumed that women feel shame and embarrassment for what they described as their disfigured faces [32]. They reported that, as women were more concerned with their beauty, they would experience the disease more negatively. In Morocco, a woman’s beauty was similarly questioned if they had CL [30]. These marks not only impacted on a girl’s beauty, they also affected her mental state. Furthermore, women with spots or marks on the face had to confront another hidden burden as the society believed these were hereditary. The fear that this mark could be passed from parent to child only existed for the potential mothers.

Similar to active lesions, scars on the face may prevent a woman from getting married as they were seen as undesirable [30–32, 40]. For instance, young women with CL scars in Morocco had fewer chances of getting married, even though they were able to work and engage in household chores. One participant noted that women with scars have a reason to fear their future due to diminished marriage prospect [31]. In this context, the stigmatised identity only extended to women and impacted mainly on unmarried women. Tellingly, none of the included studies reported men with CL being rejected for marriage or having difficulties in finding brides.

The effects on married women varied by culture. In Morocco, participants pointed out that these negative impacts occur only before marriage. If a woman developed CL after marriage and experienced subsequent scarring, then it was not grounds for a divorce as the woman became ill in her husband’s home, indicating a moral responsibility and obligation of the husband to care for his family, including his wife [31]. In other contexts, a woman’s social role within marriage was drastically affected. Afghani women with CL lesions were unable to breastfeed, touch or hug her children, and were prevented from cooking for the family [40]. The social taboos surrounding those with CL isolated the affected women in this context.

The effects from stigma represents one specific psychosocial impact that living with CL can have. Other psychosocial impacts exist in the literature. For instance, living with CL scars created additional burdens in some cultures. For example, among high school students in Errachidia Province in Morocco, scars represented a source of significant psychosocial distress [30]. While three of the male participants felt that CL negatively impacted men as it caused them to lose their masculinity, most of the participants – both male and female – felt that CL scars caused the greatest amount of psychosocial distress for girls and women. One participant equated CL scars with shame and contempt [30]. Women and girls with these CL scars report that they try to hide the scars from others. One girl commented that she had to put cream on the scar to try and mask it before she left the house [30]. Others turned to religion to cope. In this case, CL and the scars it left behind were ‘God’s will’ or ‘destiny’.

Nevertheless, demand existed for an appropriate treatment for the scars. While cosmetic surgery offered one method of reducing visible CL scars, many noted that it was neither accessible nor affordable for most. Furthermore, respondents noted that a person could experience even more mental distress after surgery as they realised that the scars would always remain [30]. Similarly, in Iran and Tunisia, cosmetic surgery was frequently mentioned in interviews. Though interviewees acknowledged that reducing the appearance of scars was more important for women and girls, particularly if they were unmarried, they highlighted financial difficulties in accessing this surgery [35]. While the lack of affordable, effective treatment preoccupied both male and female respondents, the negative psychosocial impacts were heavily skewed toward women.

In addition to stigma and distress, studies reported that fear surrounded CL. Fear of disease progression, the slow healing rate and the future implications of the disease were common amongst both men and women. In certain contexts, however, this fear manifested differently as it reflected specific social roles. Men in Colombia feared the side effects of their medication. Most were in the process of being treated with pentavalent antimonials and pentamidine isethionate and were afraid that these could leave them sterile and unable to father children in the future [34]. By contrast, women in Brazil were afraid of the physical effects of the disease itself. For them, they feared being unable to carry out the necessary household chores for which they were solely responsible [38]. These women also commented on being concerned with the perception of their bodies by other people, a concern which drove them into social isolation.

## Discussion

### The importance of gender and CL

Studies employing qualitative research methods on the neglected disease CL remain relatively sparse. Even fewer of these explore how culture impacts on understandings and perceptions of CL according to gender. The aim of this systematic literature review, then, was to explore how the cultural effects of gender affect the perception and experience of CL in the existing body of literature. In particular, we sought to establish in which ways culture impacted gender-specific health seeking behaviours; if the psychosocial impact of living with CL differed by gender; and whether stigma was experienced and, if so, whether this had a gendered dimension. The emphasis on gender recognises that women often experience differences in access to information, health services and treatment, as well as shaping vulnerability, interpretations and responses to diseases [41]. By highlighting the findings from the literature to date, we were able to better understand the existing knowledge on the intersections of gender and the neglected disease of CL.

In general, throughout the studies included in this systematic literature review, knowledge around biological aspects of CL– what it is, how it is transmitted, how to treat it, who is at risk – remained low across the different countries. Because of this, the reactions to and the implications of getting CL varied significantly across settings. While the negative psychosocial impacts of CL pervaded much of the literature, we would like to highlight that this was not uniformly experienced. For instance, many of those affected by the disease in Suriname saw no appreciable change to their lives. They conversed with friends and family, participated fully in daily life and were treated no differently [37]. The negative psychosocial impacts, therefore, are largely determined by the wider sociocultural environment in which one lives. While conditions within these social environments are the same – such as rates of poverty, access to health services, infrastructure, access to clean water and sufficient food, etc. – it must be noted that due to sociocultural factors (role expectations, available occupations, healthcare seeking norms), experiences in this social environment are different for men and women. It is for this reason that gendered differences are reported in the studies.

### Gendered dimensions of seeking healthcare

As early as the late 1990s, Vlassof and Manderson [41] published on how gender impacts on infectious diseases. They note that women often face constraints on their mobility and financial resources, opting for self-treatment or traditional healers rather than seeking formal healthcare. The data from the studies included in this systematic literature review affirm that this pattern was echoed in the literature on CL. For instance, one study found that the Ministry of Health in Colombia considered CL a health risk only for adult males due to the number of cases presenting in the clinics and hospitals [42]. However, when epidemiological surveys applied the Montenegrin test during an outbreak to detect the parasite in the general population, they found no gender differences. What was different, then, was the number of women seeking treatment for the disease. The authors posited that though women, on average, tried more treatments than men, they had less access to appropriate and effective treatments. In fact, the group of patients who reported self-medicating with Glucantime®, an approved anti-leishmanial drug, consisted almost exclusively of men. This study indicates how the gender disparities in health seeking behaviour of CL patients can lead to distortions and biases in national health planning, disease control programs and interventions at large.

These barriers to women seeking healthcare also intersect with women’s role as primary caregiver in many societies. Women often neglect to seek care, not only for financial and access reasons, but also for the social implications of doing so, particularly as giving low attention to oneself may culturally symbolise a woman’s devotion, commitment and sacrifice to family wellbeing. Furthermore, the household provision of health is predominantly the woman’s responsibility [41]. In countries such as Ecuador, this was a valued role. By seeking health care assistance outside the home, then, women felt it signified a loss of control over the patient under their care and consequently jeopardised what was seen as their culturally ascribed role as caregiver [43]. Similarly, this is why in several studies, particularly those from Costa Rica [36] and Colombia [42], women rejected the notion that one could catch CL inside the home. Catching CL inside the home indicated a failing on their part to keep their family safe and healthy. It also conferred an ‘unclean’ stigma on these women. Notably, decades have passed since these studies have taken place. These gendered norms, and their implications, may have since changed.

### Gendered impacts of stigma

It is clear that CL, in both active and inactive forms, can have significant psychosocial impacts on those living with this condition. In certain areas, the disease can cause significant stigmatisation and distress, potentially leading to social isolation or preventing the person from achieving socially valued roles such as wives and mothers. Stigma represents a social process in which a person experiences or anticipates exclusion, rejection and blame or devaluation due to a social judgment about that person or group [44]. Only five of the studies explored stigma either fully or partially and none of the studies focused on whether stigma affects men and women differently. This represents a significant gap in knowledge generation with regards to the cultural effects of gender on CL-associated stigma. This review, however, supports the idea that stigma does exist in certain contexts, and where it does, it affects men and women disproportionately, with women facing the higher burden. Two types of stigma, in particular, were mentioned in the studies. These were enacted stigma, which describes discrimination against people due to their perceived unacceptability or inferiority [45] and internalized stigma, which describes when a person accepts perceived or experienced exclusionary views and engages in self-stigmatisation [46]. For women in Morocco and Afghanistan, this created a significant barrier for marriage.

It is important to note that none of the studies discussed what the longer-term consequences could be for women who are denied the opportunity to marry because of CL-associated stigma. Even in circumstances in which both men and women experienced negative effects from the disease, it was only women who were deemed unfit for marriage due to the lesions or scars. This is a considerable gap in the literature and it makes it hard to grasp a better understanding of the full effect of CL associated stigma.

Finally, by focusing on stigma, scars and the psychosocial effects of CL, we can demonstrate the complex interwoven connections between these categories. One of the most striking and repeated findings from many of the studies was the emphasis on scars on the face. Stigma associated with scars or wounds in other places, such as the arms or legs, was not mentioned in any of the studies. This may indicate one reason why women are facing the psychosocial impact as it breaches the societal norms about women’s beauty. Because of the strong reaction to scars that was reported, we decided to dedicate a portion of our findings to this. Where the psychosocial burden of CL is greatest – such as with scars - is where we can see the stark differences in perceptions and experiences between genders emerge. Neglecting how these cultural effects impact on experiences according to gender masks the full scale of the negative implications of this disease and the scope of CL burden. Though it was a primary focus in only one of the 13 studies [30], the impacts of scarring were discussed by participants in other studies. Recent focused research suggests that the impacts of scarring has been systematically overlooked. Scars from CL (also called inactive CL) have only recently been included as a part of the disease spectrum of CL. This inclusion stems from its lasting psychosocial impact [12]. Furthermore, if the inactive form is considered, the burden of CL increases eightfold in terms of disability-adjusted life years (DALYs). From a psychosocial perspective, it has been found that inactive CL is strongly associated with social and family rejection and anxiety and depression [11]. Clearly, the negative impacts of scarring from CL warrants further research.

### Strengths and limitations

This systematic review is one of the few to tap into the rich evidence of qualitative data surrounding CL in general. Even fewer delve into the ways in which gender effects the experiences and perceptions of CL. We offer this review as a step to rectifying this gap in the literature.

As in every review, this one includes some limitations. For instance, though we made every attempt to include the various nomenclature surrounding CL, it is possible that we may have missed a key term – particularly if it was published in a non-English journal – that may have yielded more results. Furthermore, the focus on qualitative components of a study means that not only were we searching for the various forms that qualitative research can take, but we were also searching for these components within larger quantitative studies. This may have resulted in missed literature if this qualitative aspect was not clearly defined.

## Conclusions

There is some evidence that indicates that gendered differences do indeed impact on the understandings and perceptions of CL; unfortunately, many of these studies using qualitative methodology are quite old and could potentially not reflect current realities. The more recent studies devoted to this subject often rely on quantitative methods only. Quantitative methods are useful in establishing that a pattern exists, but are not sufficient to explain why a particular pattern exists. We therefore urge future research on CL to attend explicitly to the ways in which men and women in various cultural contexts experience CL, particularly as differences appear to be most pronounced in the domains of healthcare seeking, treatments, psychosocial impacts and stigma from lesions and scars. There is rich information behind the numbers that would not only greatly increase our understanding of a person’s experience with CL, but also accurately inform future programs and interventions.

## Supplementary Information

**Additional file 1.** Search strategy for Academic Search Complete.

**Additional file 2.** Search strategy for CINAHL Plus.

**Additional file 3.** Search strategy for EBSCO.

**Additional file 4.** Search strategy for EMBASE.

**Additional file 5.** Search strategy for MEDLINE.

**Additional file 6.** Search strategy for OVID.

**Additional file 7.** Search strategy for Psych Info.

## Data Availability

All data generated or analysed from this review is included in this article and its supplementary information files.

## Abbreviations

CASP: Critical appraisal skills programme
CL: Cutaneous leishmaniasis
DALYs: Disability-adjusted life years
MCL: Muco-cutaneous leishmaniasis
NTD: Neglected tropical disease
QoL: Quality of life
PICO: Participants, item of interest, comparison group, outcome of interest
PKDL: Post kala azar dermal leishmaniasis
PRISMA: Preferred reporting items for systematic reviews and meta-analyses
WHO: World Health Organization
