# Supplementary material for: Exploring the cultural effects of gender on perceptions of cutaneous leishmaniasis: a systematic literature review
Source: Glob Health Res Policy. 2022 Sep 26;7:34. doi: 10.1186/s41256-022-00266-y (PMC9511709; doi:10.1186/s41256-022-00266-y)
Supplement: Supplementary file 2 — Additional file 2. Search strategy for CINAHL Plus. [file 41256_2022_266_MOESM2_ESM.pdf]

Wednesday, July 08, 2020 9:56:51 AM

#	Query	Limiters/Expanders	Last Run Via	Results
S13	S5 AND S12	Expanders - Apply equivalent subjects<br>Search modes - Boolean/Phrase	Interface - EBSCOhost Research Databases<br>Search Screen - Advanced Search<br>Database - CINAHL Plus with Full Text	50
S12	(Spiegelberg* OR van N1 manen* OR van N1 kaam* OR merleau N1 ponty OR husserl* OR Foucault* OR Corbin* N2 strauss* OR strauss* N2 corbin* OR glaser*) OR (S6 OR S7 OR S8 OR S9 OR S10 OR S11)	Expanders - Apply equivalent subjects<br>Search modes - Boolean/Phrase	Interface - EBSCOhost Research Databases<br>Search Screen - Advanced Search<br>Database - CINAHL Plus with Full Text	1,182,752
S11	Spiegelberg* OR van N1 manen* OR van N1 kaam* OR merleau N1 ponty OR husserl* OR Foucault* OR Corbin* N2 strauss* OR strauss* N2 corbin* OR glaser*	Expanders - Apply equivalent subjects<br>Search modes - Boolean/Phrase	Interface - EBSCOhost Research Databases<br>Search Screen - Advanced Search<br>Database - CINAHL Plus with Full Text	3,217
S10	cluster sampl* OR (theme* or thematic) OR observational method* OR questionnaire* OR content analysis OR discourse* N3 analys?s OR discurs* N3 analys?s OR constant N1 comparative OR constant N1 comparison OR narrative analys?s OR Heidegger* OR Colaizzi*	Expanders - Apply equivalent subjects<br>Search modes - Boolean/Phrase	Interface - EBSCOhost Research Databases<br>Search Screen - Advanced Search<br>Database - CINAHL Plus with Full Text	726,125
S9	(action research or cooperative inquir* or co operative inquir* or co-operative inquir*) OR (humanistic or existential or experiential or	Expanders - Apply equivalent subjects<br>Search modes - Boolean/Phrase	Interface - EBSCOhost Research Databases<br>Search Screen - Advanced Search<br>Database - CINAHL Plus with Full Text	365,586

	paradigm*) OR field N1 stud* OR human science OR biographical method OR Theoretical sampl* OR Purpos* N4 sampl* OR Focus N1 group* OR (account or accounts or unstructured or open-ended or open ended or text* or narrative*) OR (life world or life-world or conversation analys?s or personal experience* or theoretical saturation) OR lived experience* OR life experience*			
S8	ethnograph* OR phenomenol* OR grounded N1 theor* OR grounded N1 study OR grounded N1 studies OR grounded N1 research OR grounded N1 analys?s OR Life stor* OR women's stor* OR (emic or etic or hermeneutic$ or heuristic$ or semiotic$) OR participant observ* OR (social construct* or postmodern* or post-structural* or post structural* or poststructural* or post modern* or post-modern* or feminis* or interpret*)	Expanders - Apply equivalent subjects<br>Search modes - Boolean/Phrase	Interface - EBSCOhost Research Databases<br>Search Screen - Advanced Search<br>Database - CINAHL Plus with Full Text	186,210
S7	MH Ethnonursing Research OR MH Constant Comparative Method OR MH Qualitative Validity+ OR MH Purposive Sampling OR MH Observational Methods+ OR MH Field Studies OR MH theoretical sample OR MH Phenomenology OR MH Phenomenological Research OR MH Life	Expanders - Apply equivalent subjects<br>Search modes - Boolean/Phrase	Interface - EBSCOhost Research Databases<br>Search Screen - Advanced Search<br>Database - CINAHL Plus with Full Text	102,636

	Experiences+ OR MH Cluster Sample+ OR Ethnonursing			
S6	MH Interview+ OR MH audiorecording OR MH Interviews+ OR MH Grounded theory OR MH Qualitative studies OR MH Research, Nursing OR MH Questionnaires+ OR MH Focus Groups OR MH Discourse Analysis OR MH Content Analysis OR MH Ethnographic Research OR MH ethnological research	Expanders - Apply equivalent subjects<br>Search modes - Boolean/Phrase	Interface - EBSCOhost Research Databases<br>Search Screen - Advanced Search<br>Database - CINAHL Plus with Full Text	748,425
S5	(DE "mucocutaneous leishmaniasis") OR (S1 OR S2 OR S3 OR S4)	Expanders - Apply equivalent subjects<br>Search modes - Boolean/Phrase	Interface - EBSCOhost Research Databases<br>Search Screen - Advanced Search<br>Database - CINAHL Plus with Full Text	550
S4	DE "mucocutaneous leishmaniasis"	Expanders - Apply equivalent subjects<br>Search modes - Boolean/Phrase	Interface - EBSCOhost Research Databases<br>Search Screen - Advanced Search<br>Database - CINAHL Plus with Full Text	0
S3	DE "cutaneous leishmaniasis"	Expanders - Apply equivalent subjects<br>Search modes - Boolean/Phrase	Interface - EBSCOhost Research Databases<br>Search Screen - Advanced Search<br>Database - CINAHL Plus with Full Text	0
S2	((TI cutaneous leishm* OR TI tegument* leishm* OR TI mucocutaneous leishm* OR TI mucosal* leishm* OR TI aleppo boil* OR TI aleppo button* OR TI aleppo sore* OR TI baghdad boil* OR TI chiclero's ulcer* OR TI oriental sore* OR TI delhi's boil*)	Expanders - Apply equivalent subjects<br>Search modes - Boolean/Phrase	Interface - EBSCOhost Research Databases<br>Search Screen - Advanced Search<br>Database - CINAHL Plus with Full Text	550

	OR (S1)) OR TI jericho button* OR TI jericho boil* OR TI jericho ulcer* OR TI "one year sore*" OR TI "one year ulcer*" OR TI "sarna brava*" OR TI "angry sore*"			
S1	TI cutaneous leishm* OR TI tegument* leishm* OR TI mucocutaneous leishm* OR TI mucosal* leishm* OR TI aleppo boil* OR TI aleppo button* OR TI aleppo sore* OR TI baghdad boil* OR TI chiclero's ulcer* OR TI oriental sore* OR TI delhi's boil*	Expanders - Apply equivalent subjects<br>Search modes - Boolean/Phrase	Interface - EBSCOhost Research Databases<br>Search Screen - Advanced Search<br>Database - CINAHL Plus with Full Text	550
