# Supplementary material for: Exploring the cultural effects of gender on perceptions of cutaneous leishmaniasis: a systematic literature review
Source: Glob Health Res Policy. 2022 Sep 26;7:34. doi: 10.1186/s41256-022-00266-y (PMC9511709; doi:10.1186/s41256-022-00266-y)
Supplement: Supplementary file 3 — Additional file 3. Search strategy for EBSCO. [file 41256_2022_266_MOESM3_ESM.docx]

| **#** | **Searches** | **Results** |
| --- | --- | --- |
| 1 | leishmaniasis OR exp leishmaniasis, cutaneous | 13,979 |
| 2 | (cutaneous* OR tegument* OR mucocutaneous OR mucosal*) | 232,724 |
| 3 | aleppo boil* | 3 |
| 4 | aleppo button* | 0 |
| 5 | aleppo ulcer* | 1 |
| 6 | aleppo sore* | 10 |
| 7 | baghdad boil* | 11 |
| 8 | chiclero's ulcer* | 10 |
| 9 | oriental sore* | 1,617 |
| 10 | delhi's boil* | 1,600 |
| 11 | Jericho button* | 0 |
| 12 | Jericho boil* | 3 |
| 13 | Jericho ulcer* | 0 |
| 14 | one year sore* | 27 |
| 15 | one year ulcer* | 357 |
| 16 | sarna brava* | 0 |
| 17 | angry sore* | 2 |
| 18 | 1 OR 2 OR 3 OR 4 OR 5 OR 6 OR 7 OR 8 OR 9 OR 10 OR 11 OR 12 OR 13 OR 14 OR 15 OR 16 OR 17 | 241,950 |
| 19 | MH Interview+ | 1,221,021 |
| 20 | MH audiorecording | 510,205 |
| 21 | MH Interviews+ | 669,874 |
| 22 | MH Grounded theory | 5,612 |
| 23 | MH Qualitative studies | **1,462,528** |
| 24 | MH Research, Nursing | 13,418 |
| 25 | MH Questionnaires+ | 91,206 |
| 26 | MH Focus Groups | 478 |
| 27 | MH Discourse Analysis | 140,673 |
| 28 | MH Content Analysis | 21,382,846 |
| 29 | MH Ethnographic Research | 43,027 |
| 30 | MH Ethnological Research | 103,488 |
| 31 | MH Ethnonursing Research | **10,375,487** |
| 32 | MH Constant Comparative Method | **1,089,743** |
| 33 | MH Qualitative Validity+ | **48,126** |
| 34 | MH Purposive Sampling | **378,311** |
| 35 | MH Observational Methods+ | **26,601,541** |
| 36 | MH Field Studies | 308,929 |
| 37 | MH theoretical sample | 2,382 |
| 38 | MH Phenomenology | 21,194 |
| 39 | MH Phenomenological Research | 11,300,397 |
| 40 | MH Life Experiences+ | **1,850,488** |
| 41 | MH Cluster Sample+ | **132,609** |
| 42 | Ethnonursing | 17,558,219 |
| 43 | ethnograph* | 23,751 |
| 44 | phenomenol* | 91,046 |
| 45 | grounded N1 theor* | 288,787 |
| 46 | grounded N1 study | 6,670,865 |
| 47 | grounded N1 studies | **12,994,884** |
| 48 | grounded N1 research | **49,673** |
| 49 | grounded N1 analys?s | **3,364** |
| 50 | Life stor* | 16 |
| 51 | women’s stor* | 13 |
| 52 | emic or etic or hermeneutic$ or heuristic$ or semiotic$ | 42 |
| 53 | participant observ* | 19 |
| 54 | social construct* or postmodern* or post-structural* or post structural* or poststructural* or post modern* or post-modern* or feminis* or interpret* | 777 |
| 55 | action research or cooperative inquir* or co operative inquir* or co-operative inquir* | 1,119 |
| 56 | humanistic or existential or experiential or paradigm* | 4,154 |
| 57 | field N1 stud* | 59 |
| 58 | field N1 research | 843 |
| 59 | human science | **17,250,822** |
| 60 | biographical method | **31,851,584** |
| 61 | Theoretical sampl* |  |
| 62 | Purpos* N4 sampl* |  |
| 63 | Focus N1 group* |  |
| 64 | account or accounts or unstructured or open-ended or open ended or text* or narrative* |  |
| 65 | life world or life-world or conversation analys?s or personal experience* or theoretical saturation |  |
| 66 | lived experience* |  |
| 67 | life experience* |  |
| 68 | cluster sampl* |  |
| 69 | theme* or thematic |  |
| 70 | observational method* |  |
| 71 | questionnaire* |  |
| 72 | content analysis |  |
| 73 | discourse* N3 analys?s |  |
| 74 | discurs* N3 analys?s |  |
| 75 | constant N1 comparative |  |
| 76 | constant N1 comparison |  |
| 77 | narrative analys?s |  |
| 78 | Heidegger* |  |
| 79 | Colaizzi* |  |
| 80 | Spiegelberg* |  |
| 81 | van N1 manen* |  |
| 82 | van N1 kaam* |  |
| 83 | merleau N1 ponty* |  |
| 84 | husserl* |  |
| 85 | Foucault* |  |
| 86 | Corbin* N2 strauss* |  |
| 87 | strauss* N2 corbin* |  |
| 88 | glaser* |  |
| 89 | 18 and 89 | 184,752 |
