# Supplementary material for: Exploring the cultural effects of gender on perceptions of cutaneous leishmaniasis: a systematic literature review
Source: Glob Health Res Policy. 2022 Sep 26;7:34. doi: 10.1186/s41256-022-00266-y (PMC9511709; doi:10.1186/s41256-022-00266-y)
Supplement: Supplementary file 5 — Additional file 5. Search strategy for MEDLINE. [file 41256_2022_266_MOESM5_ESM.docx]

Database(s): **Ovid MEDLINE(R) and In-Process & Other Non-Indexed Citations**1946 to July 03, 2020
Search Strategy:

| **#** | **Searches** | **Results** |
| --- | --- | --- |
| 1 | leishmaniasis/ or exp leishmaniasis, cutaneous/ | 14456 |
| 2 | ((cutaneous* or tegument* or mucocutaneous or mucosal*) adj5 leishm*).ti,ab,kf. | 8902 |
| 3 | aleppo boil*.ti,ab,kf. | 15 |
| 4 | aleppo button*.ti,ab,kf. | 0 |
| 5 | aleppo ulcer*.ti,ab,kf. | 1 |
| 6 | aleppo sore*.ti,ab,kf. | 0 |
| 7 | baghdad boil*.ti,ab,kf. | 5 |
| 8 | chiclero's ulcer*.ti,ab,kf. | 21 |
| 9 | oriental sore*.ti,ab,kf. | 137 |
| 10 | delhi's boil*.ti,ab,kf. | 0 |
| 11 | Jericho button*.ti,ab,kf. | 0 |
| 12 | Jericho boil*.ti,ab,kf. | 2 |
| 13 | Jericho ulcer*.ti,ab,kf. | 0 |
| 14 | one year sore*.ti,ab,kf. | 1 |
| 15 | one year ulcer*.ti,ab,kf. | 2 |
| 16 | sarna brava*.ti,ab,kf. | 0 |
| 17 | angry sore*.ti,ab,kf. | 0 |
| 18 | 1 or 2 or 3 or 4 or 5 or 6 or 7 or 8 or 9 or 10 or 11 or 12 or 13 or 14 or 15 or 16 or 17 | 16409 |
| 19 | Qualitative Research/ | 55100 |
| 20 | interview/ | 29160 |
| 21 | (theme* or thematic).mp. | 109660 |
| 22 | qualitative.af. | 248059 |
| 23 | nursing methodology research/ | 16373 |
| 24 | questionnaire*.mp. | 742229 |
| 25 | ethnological research.mp. | 7 |
| 26 | ethnograph*.mp. | 10967 |
| 27 | ethnonursing.af. | 116 |
| 28 | phenomenol*.af. | 26679 |
| 29 | (grounded adj (theor* or study or studies or research or analys?s)).af. | 11908 |
| 30 | (life stor* or women* stor*).mp. | 1371 |
| 31 | (emic or etic or hermeneutic* or heuristic* or semiotic*).af. | 17889 |
| 32 | ((data adj1 saturat*) or participant observ*).tw. | 5746 |
| 33 | (social construct* or postmodern* or post modern* or poststructural* or post structural* or feminis* or interpret*).mp. | 528415 |
| 34 | (action research or cooperative inquir* or co operative inquir*).mp. | 4289 |
| 35 | (humanistic or existential or experiential or paradigm*).mp. | 154863 |
| 36 | (field adj (study or studies or research)).tw. | 16454 |
| 37 | human science.tw. | 245 |
| 38 | biographical method.tw. | 17 |
| 39 | theoretical sampl*.af. | 697 |
| 40 | ((purpos* adj4 sampl*) or (focus adj group*)).af. | 65327 |
| 41 | (account or accounts or unstructured or open ended or text* or narrative*).mp. | 645156 |
| 42 | (life world or conversation analys?s or personal experience* or theoretical saturation).mp. | 15270 |
| 43 | ((lived or life) adj experience*).mp. | 11586 |
| 44 | cluster sampl*.mp. | 7472 |
| 45 | observational method*.af. | 772 |
| 46 | content analysis.af. | 28222 |
| 47 | (constant adj (comparative or comparison)).af. | 4641 |
| 48 | ((discourse* or discurs*) adj3 analys?s).tw. | 2234 |
| 49 | narrative analys?s.af. | 1271 |
| 50 | heidegger*.tw. | 655 |
| 51 | colaizzi*.tw. | 705 |
| 52 | spiegelberg*.tw. | 80 |
| 53 | van manen*.tw. | 418 |
| 54 | van kaam*.tw. | 39 |
| 55 | merleau ponty.tw. | 148 |
| 56 | husserl*.tw. | 248 |
| 57 | foucault*.tw. | 803 |
| 58 | (corbin* adj2 strauss*).tw. | 334 |
| 59 | glaser*.tw. | 991 |
| 60 | (mix* adj2 (method* or design*)).af. | 29444 |
| 61 | 19 or 20 or 21 or 22 or 23 or 24 or 25 or 26 or 27 or 28 or 29 or 30 or 31 or 32 or 33 or 34 or 35 or 36 or 37 or 38 or 39 or 40 or 41 or 42 or 43 or 44 or 45 or 46 or 47 or 48 or 49 or 50 or 51 or 52 or 53 or 54 or 55 or 56 or 57 or 58 or 59 or 60 | 2321395 |
| 62 | 18 and 61 | 634 |
