# Supplementary material for: Exploring the cultural effects of gender on perceptions of cutaneous leishmaniasis: a systematic literature review
Source: Glob Health Res Policy. 2022 Sep 26;7:34. doi: 10.1186/s41256-022-00266-y (PMC9511709; doi:10.1186/s41256-022-00266-y)
Supplement: Supplementary file 6 — Additional file 6. Search strategy for OVID. [file 41256_2022_266_MOESM6_ESM.docx]

Database(s): **Ovid MEDLINE(R) and In-Process & Other Non-Indexed Citations**1946 to June 03, 2020
Search Strategy:

| **#** | **Searches** | **Results** |
| --- | --- | --- |
| 1 | leishmaniasis/ or exp leishmaniasis, cutaneous/ | 14453 |
| 2 | ((cutaneous* or tegument* or mucocutaneous or mucosal*) adj5 leishm*).ti,ab,kf. | 8895 |
| 3 | aleppo boil*.ti,ab,kf. | 15 |
| 4 | aleppo button*.ti,ab,kf. | 0 |
| 5 | aleppo ulcer*.ti,ab,kf. | 1 |
| 6 | aleppo sore*.ti,ab,kf. | 0 |
| 7 | baghdad boil*.ti,ab,kf. | 5 |
| 8 | chiclero's ulcer*.ti,ab,kf. | 21 |
| 9 | oriental sore*.ti,ab,kf. | 137 |
| 10 | delhi's boil*.ti,ab,kf. | 0 |
| 11 | Jericho button*.ti,ab,kf. | 0 |
| 12 | Jericho boil*.ti,ab,kf. | 2 |
| 13 | Jericho ulcer*.ti,ab,kf. | 0 |
| 14 | one year sore*.ti,ab,kf. | 1 |
| 15 | one year ulcer*.ti,ab,kf. | 2 |
| 16 | sarna brava*.ti,ab,kf. | 0 |
| 17 | angry sore*.ti,ab,kf. | 0 |
| 18 | 1 or 2 or 3 or 4 or 5 or 6 or 7 or 8 or 9 or 10 or 11 or 12 or 13 or 14 or 15 or 16 or 17 | 16401 |
| 19 | Social support/ | 70330 |
| 20 | Experience*.ti,ab. | 1068229 |
| 21 | Perception*.ti,ab. | 249509 |
| 22 | Econ*.ti,ab. | 292532 |
| 23 | Finance*.ti,ab. | 11275 |
| 24 | Health*.ti,ab. | 2714164 |
| 25 | Stigma*.ti,ab. | 36062 |
| 26 | Quality of life*.ti,ab. | 276074 |
| 27 | (mental adj3 health*).ti,ab. | 148152 |
| 28 | Cop*.ti,ab. | 487910 |
| 29 | Psych*.ti,ab. | 811716 |
| 30 | Psychosocial*.ti,ab. | 94002 |
| 31 | Support*.ti,ab. | 1535189 |
| 32 | Mood*.ti,ab. | 74540 |
| 33 | Treatment*.ti,ab. | 4474678 |
| 34 | Care*.ti,ab. | 1635601 |
| 35 | Healthcare*.ti,ab. | 210280 |
| 36 | Anxi*.ti,ab. | 206049 |
| 37 | Depress*.ti,ab. | 456117 |
| 38 | Well being.ti,ab. | 75233 |
| 39 | Wellbeing.ti,ab. | 15668 |
| 40 | Health seeking.ti,ab. | 2583 |
| 41 | Healthseeking.ti,ab. | 5 |
| 42 | 19 or 20 or 21 or 22 or 23 or 24 or 25 or 26 or 27 or 28 or 29 or 30 or 31 or 32 or 33 or 34 or 35 or 36 or 37 or 38 or 39 or 40 or 41 | 9938352 |
| 43 | 18 and 42 | 6038 |
| 44 | (mix* adj2 (method* or design*)).af. [filter amended to identify mixed method studies] | 29390 |
| 45 | 43 and 44 | 4 |
| 46 | Gender identity/ph [Physiology] | 5 |
| 47 | Sex.ti,ab.kf. | 520801 |
| 48 | Sex difference*.ti,ab,kf. | 35530 |
| 49 | Sex distribution*.ti,ab,kf. | 5227 |
| 50 | Sex character*.ti,ab,kf. | 2111 |
| 51 | Sex based.ti,ab,kf. | 1649 |
| 52 | Sex factor*.ti,ab,kf. | 2870 |
| 53 | Sex dimorphism*ti,ab,kf. | 575 |
| 54 | Gender diferenc*.ti,ab,kf. | 32237 |
| 55 | Gender based*.ti,ab,kf. | 3330 |
| 56 | (gender or sex).ti,ab. And human*. | 622956 |
| 57 | 46 or 47 or 48 or 49 or 50 or 51 or 52 or 53 or 54 or 55 or 56 | 764864 |
| 58 | 43 and 57 | 134 |
| 59 | Limit 58 to humans | 103 |
| 60 | Limit 43 to humans | 3831 |
| 61 | 18 and 44 | 6 |
| 62 | (leishm* not post kala) | 37300 |
| 63 | Gender.ti,ab,kf | 317922 |
| 64 | 61 and 63 | 55 |
| 65 | 62 and 64 | 50 |
| 66 | Limit 65 to humans | 36 |
